# Online HIV prevention intervention on condomless sex among men who have sex with men: a web-based randomized controlled trial

**DOI:** 10.1186/s12879-019-4251-5

**Published:** 2019-07-19

**Authors:** Weibin Cheng, Huifang Xu, Weiming Tang, Fei Zhong, Gang Meng, Zhigang Han, Jinkou Zhao

**Affiliations:** 1 Department of HIV/AIDS Control and Prevention, Guangzhou Center for Disease Control and Prevention, No.1, Qide Road, Baiyun District, Guangzhou, 510440 Guangdong China; 2 Dermatology Hospital of Southern Medical University, No.2 Lujing Road, Yuexiu District, Guangzhou, 510095 Guangdong China; 3 University of North Carolina Project-China, No.2 Lujing Road, Yuexiu District, Guangzhou, 510095 Guangdong China; 4 Lingnan Partners Community Support Center A2-Tianwenyuan, Tiyuxi road, Guangzhou, 510000 Guangdong China; 5 The Global Fund to fight AIDS, Tuberculosis and Malaria, Chemin de Blandonnet 8 | 1214 Vernier, Geneva, Switzerland

**Keywords:** Human immunodeficiency virus, Intervention, Condomless sex, Men who have sex with men, Internet, Randomized controlled trial

## Abstract

**Background:**

Given the widespread use of the Internet among men who have sex with men (MSM) and high risk of Internet-facilitated sexual behaviors, Internet-based interventions to reduce sexual risk are urgently needed.

**Methods:**

We recruited 1,100 participants from online and randomly assigned to two groups. One group received online HIV intervention services. Online HIV intervention was developed through mix-method formative research, measures included scenarios experiencing intervention and HIV information dissemination. Self-reported condomless anal sex with a male in the past three months was measured to evaluate the intervention effect.

**Result:**

Of the 1,100 participants, the majority were aged between 21 and 30 years old (62%), had a college degree or higher (80%), were single (88%), and self-identified as homosexual (78%). The estimated risk difference of condomless sex with a male in the past three months between groups was 9.3% (95% confidence interval (CI): 1.1, 17.5%). Using multiple imputations intention-to-treat, the estimated risk difference was 8.9% (95%CI: 1.2, 16.6%). Modification effects were found between intervention and characteristics including: educational attainment (*p* = 0.012), marital status (*p* = 0.005) and awareness of AIDS-related knowledge (*p* = 0.010).

**Conclusion:**

Internet appears to be a promising approach to disseminate HIV prevention amongst MSM. Interactive online intervention appeals to MSM and poses a great potential for reducing HIV risky behavior.

**Trial registration:**

ChiCTR1800014260 (retrospectively registered 2 Jan, 2018).

**Electronic supplementary material:**

The online version of this article (10.1186/s12879-019-4251-5) contains supplementary material, which is available to authorized users.

## Background

The expanding HIV epidemic among men who have sex with men (MSM) is one of the most pressing challenges regarding HIV transmission in China [1]. Since 2011, HIV transmission through male to male sex has become the second most common transmission route among new HIV infections [2, 3]. The persistent high HIV incidence has contributed to a continuingly rising HIV prevalence from 2.5% in 2006 to 7.4% in 2011 [4–7]. In Guangzhou, our MSM cohort data also showed a high HIV incidence rate among MSM, estimated at an average of 5.6 per 100 person-years (95% confidence interval (CI): 4.4–7.0) from 2008 to 2012 [8]. The HIV prevalence rate rose from 5.2% in 2008 to 12.3% in 2014 among MSM in Guangzhou [9]. Identifying and implementing effective intervention strategies is therefore urgently required to prevent further spread of the HIV epidemic among MSM in China.

Growing evidence shows that the Internet has emerged as the most popular platform for facilitating sex networking among MSM [10–14]. The Internet, in combination with the wide use of mobile phones, has revolutionized MSM networking globally, which has facilitated the spread of HIV infections. Studies have shown that Internet-facilitated sexual encounters are associated with high-risk behaviors related to HIV and sexual transmitted infections (STIs) [15]. Compared to MSM who sought sex partners traditionally (i.e. bars, clubs, parks, and saunas), MSM who sought sex partners online were more likely to have condomless anal sex [13, 15–21]. Given the wide spread of Internet use among MSM and risk of HIV transmission through Internet-facilitated sexual behaviors, effective online interventions need to be explored and implemented [22]. Other online interventions have used several different measures, including dissemination of prevention messages [23], using visual stories for health education [24], developing gay avatars on a virtual cruise game to simulate risk of HIV infection [25], and applying behavioral theory to design prevention programmes [26], to reduce risk behaviors and/or to promote HIV testing. Unfortunately, many of the trials have failed to achieve significant effects. High attrition rates have prevented meaningful interpretation of results [23, 24]. Nonetheless, researchers have noted that Internet-based interventions are likely to have significant effects but more effective methods are needed to conduct and to evaluate Internet-based interventions.

In 2010, under the support of the China—Gate HIV Prevention Program, we developed an interactive computer programme (details given in methods section) which aimed to reduce HIV-related risk behaviors among MSM who have frequent use of the Internet. In order to examine the efficacy of the intervention in promoting safe sex behaviors, we conducted a randomized controlled trial online among Chinese MSM.

## Methods

### Study design

This randomized controlled trial was conducted between September 2010 and June 2011. Trial implementation and reporting adhered to CONSORT guidelines. A CONSORT checklist of information is provided as a Additional file 1. This trial was implemented on China’s earliest and Southern China’s largest gay website. This website was very popular among the gay community and attracted 483,844 unique visits across China (statistics by Google Analytics) and retained over 100,000 registered members in 2012 (website statistics). The eligibility of target participants of this study was Internet users who were male, aged 18 years old or above, had been engaged in sexual intercourse with other men six months prior to the study, and agreed to use the same account for the next six months. Participants were excluded if they have participated in an HIV intervention study before. Recruitment advertisements were placed on the web portal and posted at the top of the website bulletin board system (BBS). Visitors who were interested in the study clicked on the advertisement and were linked to the survey platform. A consent script with detailed study information was shown on the welcome page. Consents were obtained by using an online ‘click to consent’ procedure. Participants were instructed to click to confirm their consent if they agreed to participate in the study. Participants who consented were then directed to the screening questionnaire. Eligible participants were directed to the baseline survey. A unique registered name was used as an identifier to prevent repeated participation and to track for follow-up. Recruitment closed when the required sample size was reached.

In this study, sample size was estimated based on the change in condomless anal intercourse with male partners. Though there was no available effect size reference data, we expected that the intervention should bring down the proportion of condomless anal intercourse at least 10% to have practical application significance. In some previous studies, due to excessive loss of follow-up, the power of the statistical analysis was diminished. In this trial, loss to follow-up was estimated based on the website registered user’s active rate in three months. The active rate reflected that 85% of the registered users logged on at least once in three months interval. Thus, we expected 15% of participants might loss to follow-up. Finally, we estimated condomless sex in the past three months was 40% (P_1_), based on a survey conducted in 2008 [10]. Assuming that condomless anal sex would be stable in the control group, while brought down to 30% in the intervention group at six months follow-up (P_2_). With a marginal error of 5% (alpha), statistical power of 0.8, and taking 15% loss to follow-up into consideration, the required sample size would be 404 for each group. After taking possible subgroup analyses into consideration, we expected to recruit 550 MSM in each group.

Participants who completed the baseline and six-month post-survey were rewarded a virtual community gift as compensation for their time. The virtual community gift included BBS credits and prestige points. Credits were a token of virtual currency in the BBS that can be used for buying virtual community goods, such as followers, eggs and gift cards, etc. Prestige points were related to grade level of the member; the higher grade level grants more permissions, such as lookup member profiles and downloading and uploading files in the BBS. The study protocol was approved by the Ethics Committee of Guangzhou Center for Disease Control and Prevention.

### Randomisation and masking

This study was a non-blinded design due to the nature of the intervention. Upon the completion of informed consent and eligibility criteria screening, a preset computer randomisation algorithm was used to assign the participants into either the intervention or control group with a ratio of 1:1. The intervention group was provided with the intervention service described below, while the control group received none of the prescribed intervention measures. Both groups were provided the standard HIV referral service, which was to recommend participants take an HIV test at a local clinic. Interventions delivery and data collection were completed online.

### Intervention measures

#### Formative research

In order to learn the barriers and facilitators for online HIV services among MSM, we conducted a mixed method research which included a brief quantitative online survey and a structured qualitative interview on the study website. Quantitative results showed that 84.7% of MSM preferred to obtain HIV information through the Internet. More detailed information of formative research findings is given in our previous published article [27]. Structured online interviews among 574 gay men suggested that ignorance of the risk of HIV transmission and misconceptions about risk behaviors were the major reasons for condomless anal sex.

Based upon the formative research results, we developed the intervention measure used in this study. Intervention materials were delivered in Chinese. It included part I, an interactive design of scenarios experiencing interventions, called “Choice of Life”, and part II, HIV information dissemination, named “Health Messenger” (detailed below). This intervention engaged participants in real scenarios by presenting peer attitudes towards behavioral decisions, and subsequently releasing HIV epidemic data and related knowledge to strengthen participants’ HIV risk perception, thus encouraging behavior change [28]. A multidisciplinary team of health professionals, social scientists, computer specialists and community experts reviewed the intervention. Interventions were sent directly to the users’ account, formats including news alert showed on log-on page and message in the mail box.

#### Part I: scenarios experiencing intervention (choice of life)

This intervention was delivered to the intervention group immediately after completion of baseline survey. An interactive dialogue box with a new message sign was tagged on the private zone of the participants’ log-in page, which made it easily noticed when participants logged in. The dialogue box lasted for seven days until it had been clicked and the process was completed. The process began with a monologue, for example ‘I broke up with my man … ’, then solicited to click to further enter into the story. After that, a contextualized option popped-up and asked for participants to make a decision. In the end, participants were told what happened in the story, and what decision had been made by those in the story and other participants (see Additional file 2). Stories were solicited from the gay community and reviewed by the research team and target population. There were five scenarios, which included having unprotected anal intercourse with an intimate partner, encountering a sex partner in the pub, having sex with a commercial sex partner, experiencing a broken condom during intercourse, and taking an HIV test.

#### Part II: HIV information dissemination (health messenger)

Three themes of HIV information were elaborated upon and tailored for MSM and made visually appealing as well as MSM friendly (see Additional files 3). One theme each week was sent directly to the participants’ e-mail address at the end of part I. Theme I, named “know more & love yourself more”, delivered basic knowledge of HIV/AIDS and risky contact for HIV transmission. Theme II, named “risky domino, which one is you?”, released the latest local HIV epidemic data among MSM to draw attention to risk awareness. Theme III, named “love faithfully & bottom safety”, clarified the misconceptions of sex behaviors, especially in intimate relationships.

This intervention package was focused on the four key determinants of health-related behaviors: attitudes, subjective norms, perceived control, and behavioral intention, as identified by the Theory of Planned Behavior [29]. With the aims of addressing ignorance and misconceptions, participants experiencing the intervention were presented real-life scenarios to increase HIV risk perceptions and shared peers’ view to generate community norms awareness. The subsequent officially released HIV epidemic information materials further strengthened perception of consequences of practicing condomless anal sex to further enhance safe sex behavior.

### Outcome measures

The primary outcome of this trial was self-reported condomless anal sex with another male in the past three months. Given the discrepancy of condom use between regular and casual partners, we conducted a subgroup analysis of condomless sex by types of sex partner. Condomless anal sex was categorized as 1) a condom was not used all the time during anal intercourse with males in the past three months 2) No anal intercourse with males in the past three months and 3) condoms were used every time during anal sex (defined as no condomless anal sex).

### Statistical analysis

We examined the hypothesis (Wald method) that the Internet intervention was more effective than the standard referral service in promoting safe sex behavior by looking at the differences in proportions of condomless anal sex between the two groups. Effect modification was assessed using a linear probability model [30] based on demographics and the pre-specified subgroup of awareness of AIDS-related knowledge (defined by answering 6 or more questions correctly in an 8-items scale), measured at baseline.

Demographics and HIV-related behaviors were compared for participants who responded to the post-survey and those who did not. The primary analysis included only individuals who responded to the post-survey, i.e., a completed record analysis. Intention-to-treat (ITT) with multiple imputations, which was used to impute the missing responses at post-survey, was used as a sensitivity analysis. Predictors in the imputation model included information collected only at baseline and at both time points. Baseline information included age, educational attainment, income, marital status, ethnicity, sexual orientation, places of meeting sex partners, HIV test history, and AIDS-related knowledge. Information captured at both time points included perception of HIV epidemic, sexual behaviors (group sex, condomless sex), and number of sexual partners in the past three months. Statistical analysis was performed using IBM SPSS Statistic Software for Windows Version 18 (SPSS Inc., Chicago, USA) and *P* < 0.05 was considered to be statistically significant. This trial was retrospectively registered with the Chinese Clinical Trial Registry, number ChiCTR1800014260.

## Results

**Fig. 1:**
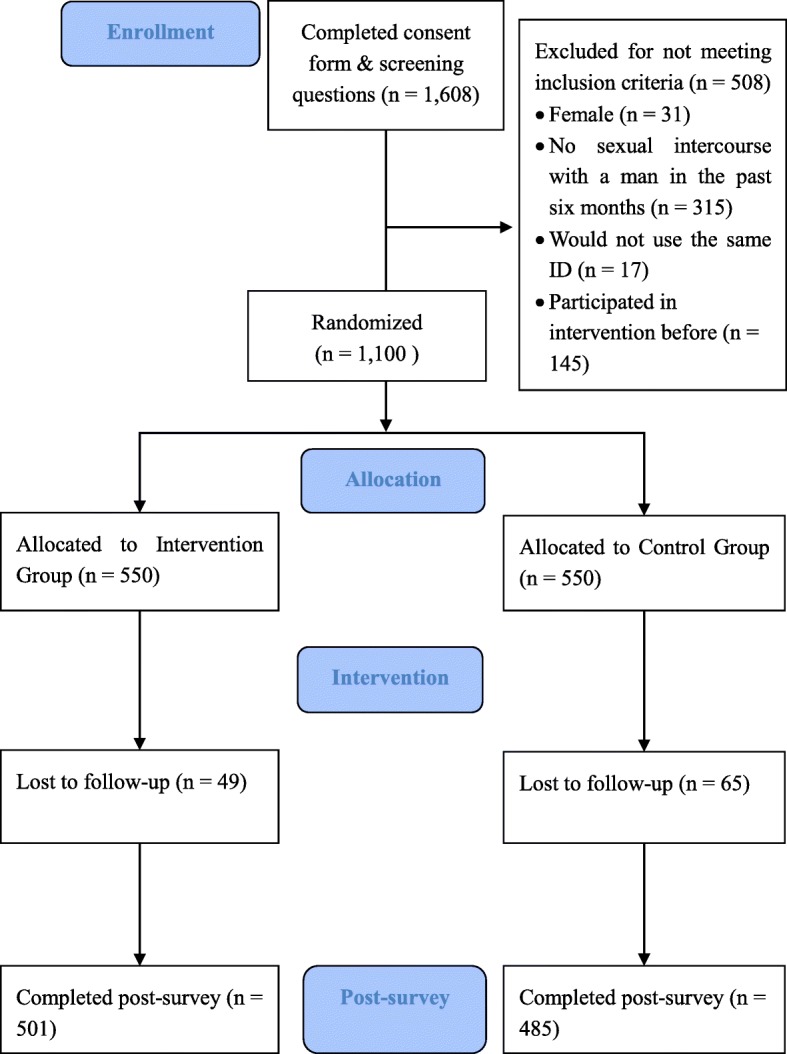
Flow of recruitment, randomisation, and follow-up. In this flowchart, we provide detailed information on trial participants’ enrollment, eligibility criteria screening, randomisation results, and intervention delivery

Figure 1 presents the enrollment, allocation, and retention rates in the different groups in this study. Overall, a total of 1,608 participants were recruited and completed the baseline screening procedure. After the baseline survey, a total of 1,100 eligible participants were randomly allocated into either intervention or control group. In the intervention group, 501 participants (91.09%) received the intervention measures and responded to the post-survey, while 49 participants were lost to follow up. In the control group, 485 participants (88.18%) responded to the post-survey, while 65 participants were lost to follow up. There was no significant difference in the retention rate between the two groups (*χ*^2^ = 2.51, *P* = 0.11).

Table 1 presents the socio-demographic and behavioral characteristics of the participants in both intervention and control groups at baseline. Of the 1,100 participants, the majority were aged between 21 and 30 years old (62%), had a college degree or higher (80%), were single (88%), self-identified as homosexual (78%), and were meeting sex partners mainly through the Internet (88%). One quarter of participants had tested for HIV in the previous six months. The majority of participants had male sex partner/s in the past three months (75%), and 68% had regular sex partner/s, 55% had casual sex partner/s. Overall, 46% of the participants reported ever having had condomless anal sex with their male partner/s. The randomisation procedure achieved a balance of demographics and behavior characteristics between the two groups (Table 1).

**Table 1:** Baseline demographic and behavioral characteristics of study participants recruited and randomized online in China, 2011 (*n* = 1,100)

Characteristic	Standard referral (*N* = 550)-No (%)	Online Intervention (*N* = 550)-No (%)
Age –Years		
≤ 20	32 (5.8)	24 (4.4)
21–30	336 (61.1)	351 (63.8)
31–40	150 (27.3)	137 (24.9)
≥ 41	32 (5.8)	38 (6.9)
Educational		
Junior high school or below	20 (3.6)	17 (3.1)
Senior High school	102 (18.5)	84 (15.3)
College or above	428 (77.8)	449 (81.6)
Annual income (US dollar)		
No income	49 (8.9)	37 (6.7)
Less than $5,351	204 (37.1)	160 (29.1)
$5,351 to 12,485	198 (36.0)	242 (44.0)
$12,485 or above	99 (18.0)	111 (20.2)
Ethnicity		
Han	539 (98.0)	531 (96.5)
Minority	11 (2.0)	19 (3.5)
Marital status		
Currently married	78 (14.2)	56 (10.2)
Single^a^	472 (85.8)	494 (89.8)
Sexual orientation		
Homosexual	428 (77.8)	429 (78.0)
Bisexual/heterosexual/other	122 (22.2)	121 (22.0)
Places of meeting sex partner/s		
Internet	488 (88.7)	480 (87.3)
Other	62 (11.3)	70 (12.7)
Perception of HIV epidemic among MSM		
Nothing serious	191 (34.7)	195 (35.5)
Serious/very serious	359 (65.3)	355 (64.5)
HIV tested in the previous six months		
Yes	148 (26.9)	126 (22.9)
No	402 (73.1)	424 (77.1)
Had anal sex with male in the previous three months		
Yes	427 (77.6)	399 (72.5)
No	123 (22.4)	151 (27.5)
Group sex with male in the previous three months		
Yes	76 (13.8)	67 (12.2)
No	474 (86.2)	483 (87.8)
Condomless anal sex with male in the previous three months (overall condomless sex)		
Yes	265 (48.2)	243 (44.2)
No	285 (51.8)	307 (55.8)
Condomless anal sex with male regular partner/s in the previous three months		
Yes	213 (54.9)	192 (53.3)
No	175 (45.1)	168 (46.7)
Condomless anal sex with male casual partner/s in the previous three months		
Yes	109 (34.1)	105 (37.0)
No	211 (65.9)	179 (63.0)

^a^Includes divorced and widowed

*MSM* men who have sex with men

There were no significant differences between the 986 participants who responded to the post-survey and those who were lost to follow up (see Additional file 4). In the post-survey, the proportion of participants who had had condomless anal sex in the past three months was 47.8% in the control group and 38.5% in the intervention group, respectively. For the completed-records analysis the estimated risk difference between groups was 9.3% (95%CI: 1.1, 17.5%). Using multiple imputations intention-to-treat, the estimated risk difference was 8.9% (95%CI: 1.2, 16.6%) (Table 2). The subgroup analysis results showed in both the control group and intervention group, condomless sex with male regular partner/s was high, compared with condomless sex with male casual partner/s (53.4% versus 38.9% in the control group, 46.9% versus 29.7% in intervention group). However, the estimated risk difference between groups was not significant in either case:(6.5% (95%CI: − 4.4, 17.3%) among condomless sex with regular partner; 9.2% (95%CI: − 1.3, 19.6%) among condomless sex with casual partner) (Table 2).

**Table 2:** Efficacy of the online intervention in reducing condomless anal sex among Chinese MSM, 2011 (n = 1,100)

	Standard referral– No (%)	Online Intervention – No (%)	Difference in proportions (%)	95% CI (%)
Completed-records analysis (*n* = 986)				
Overall condomless sex in the previous three months	232 (47.8)	193 (38.5)	9.3	(1.1, 17.5)
Multiple imputation ITT analysis (n = 1,100)				
Overall condomless sex in the previous three months	258 (46.9)	209 (38.0)	8.9	(1.2, 16.6)
Subgroup analysis				
Condomless sex with male regular partner/s in the previous three months	183 (53.4)	144 (46.9)	6.5	(−4.4, 17.3)
Condomless sex with male casual partner/s in the previous three months	98 (38.9)	68 (29.7)	9.2	(−1.3, 19.6)

*MSM* men who have sex with men, *CI* confidence interval, *ITT* intention-to-treat

We assessed the differences in proportions of condomless sex between the intervention and control groups by testing for effect modification (Table 3). Effect modification was found between the intervention and educational attainment (*p* = 0.012), marital status (*p* = 0.005) and awareness of AIDS-related knowledge (*p* = 0.010). Intervention efficacy was significant in participants who were single (risk difference: 9.2% (95%CI: 0.5, 18.0%)), who had an annual income less than $5,351 (risk difference: 15.5% (95%CI: 1.8, 29.3%)), who met sex partners through the Internet (risk difference: 10.8% (95%CI: 2.1, 19.5%)), and had high awareness of AIDS-related knowledge (risk difference: 9.4% (95%CI: 1.0, 17.9%)) (Table 3).

**Table 3:** Subgroup analyses of online intervention in randomized controlled trial in China, 2011

Subgroup	Standard referral *n/N (%)*	Online Intervention *n/N (%)*	Difference in proportions % (95% CI)	*P* value for interaction
Educational attainment				
Middle school or lower	9/19 (47.4)	5/14 (35.7)	11.7 (−32.4, 55.7)	0.012
High school	45/94 (47.9)	29/76 (38.2)	9.7 (−10.0, 29.4)	–
College or above	178/372 (47.8)	159/411 (38.7)	9.1 (−0.1, 18.4)	–
Marital status				
Currently married	34/69 (49.3)	18/45 (40.0)	9.3 (−15.5, 34.1)	0.005
Single^a^	198/416 (47.6)	175/456 (38.4)	9.2 (0.5, 18.0)	–
Annual income (US dollar)				
No income	16/48 (33.3)	15/35 (42.9)	−9.6 (−36.7, 17.6)	0.445
Less than $5,351	89/183 (48.6)	48/145 (33.1)	15.5 (1.8, 29.3)	–
$5351 to 12,485	82/167 (49.1)	82/220 (37.3)	11.8 (−1.5, 25.2)	–
$12,485 or above	45/87 (51.7)	48/101 (47.5)	4.2 (−16.0, 24.4)	–
Places of meeting sex partner				
Internet	209/432 (48.4)	165/439 (37.6)	10.8 (2.1, 19.5)	0.092
Other	23/53 (43.4)	28/62 (45.2)	−1.8 (−26.1, 22.6)	–
Awareness of AIDS-related knowledge				
Yes	219/456 (48.0)	183/474 (38.6)	9.4 (1.0, 17.9)	0.010
No	13/29 (44.8)	10/27 (37.0)	7.8 (−25.7, 41.3)	–

^a^Includes divorced and widowed

## Discussion

Our study demonstrated that using the Internet to disseminate HIV prevention messages could reduce risky sex behaviors among MSM. Both completed-records and multiple imputations intention-to-treat results showed that the intervention had significantly lower levels of condomless anal sex compared to the control group. Internet mediated HIV intervention appears to be a promising approach for HIV prevention amongst MSM [31, 32]. This randomized controlled trial extends evidence on used of message dissemination and Internet-based intervention to intervene risk sex behaviors among MSM [24, 25]. The effect most possible attributes to use of the advanced Internet technology so as to present HIV prevention intervention in an interactive way, which participants took part in the intervention proactively [33]. Though intervention content was community-contextualized and specific, this is a simple, straight-forward design which is replicable and easily scaled up for other Internet settings both domestically and internationally.

Our sub-analyses showed that the effect of the intervention was modified by socioeconomic background. More prominent efficacy was found among participants who had higher educational attainment, were single, and had less income. Past studies suggested that lower socioeconomic status MSM were disproportionately affected by HIV [6, 34]. Meanwhile, same-sex marriage isn’t legal in China, MSM who are marred to women may have less access to HIV care services [6, 35]. However, our intervention failed to achieve effects in these subgroups. Intervention research targeting vulnerable MSM subgroups in greatly needed.

Intervention measures design were focused on addressing ignorance and misconceptions of HIV transmission among MSM. We paid special attention to the realities and complexity of sexual behaviors and presented real-life scenarios in an interactive way which is promising in increasing HIV risk perceptions among MSM [24, 26, 33, 36]. Significant changes were also observed in attitudes and behavioral intentions between baseline and six months follow-up within the intervention group (see Additional file 4). Meanwhile, formative research identified a simple interactive way to get participants involved in the intervention proactively [32, 37–41]. Furthermore, intervention measures were delivered directly to the users’ account (log-on page and E-mail box) confidentially, which reduced the possibility of between groups contamination.

It should be noted that this study has examined voluntary participants recruited from registered members of one gay web portal; the characteristics of the participants may differ from other non-registered MSM. Also, the information collected in this trial was based on self-report, which may be prone to social desirability [42], although this may have been mitigated by the computer-aided self-administered online survey to some extent. In addition, participants may have been subjected to other interventions both online and offline, and the effect of such exposure cannot be quantified. However, we assumed this marginal effect between the two groups was comparable. Lastly, the randomized controlled trial only lasted for six months so the long-term effect of the intervention is still unknown.

## Conclusions

Notwithstanding its limitations, our online intervention was associated with 9% fewer reports of condomless sex compared to the standard referral service. This community engaged and user-favorite intervention design may be applicable and scalable in other Internet settings. Further research observing objective indicators, such as how effectively online interventions convert to offline HIV testing and reduce HIV/STIs incidence, is still required.

## Additional files

Additional file 1: CONSORT 2010 checklist of information to include when reporting a randomised trial. (DOC 218 kb)

Additional file 2: Screenshots of the Scenario Experiencing Intervention. (DOCX 4707 kb)

Additional file 3: Screenshots of the Health Messenger. (DOCX 301 kb)

Additional file 4: Comparison of participants’ baseline sociodemographic characteristics in completed records versus lost to follow-up and participants’ baseline and post-survey measurements on HIV/AIDS-related attitudes and behavioral intention in intervention group (n=501) versus control group. (DOCX 20 kb)

## Data Availability

The datasets generated and analyzed during the current study are available in the WHO International Clinical Trials Registry Platform of Chinese Clinical Trial Registry (ChiCTR) (http://www.chictr.org.cn/showprojen.aspx?proj=18197). Other materials are available from the corresponding author on reasonable request.

## Abbreviations

AIDS: Acquired immunodeficiency syndrome
BBS: Bulletin board system
CI: Confidence interval
HIV: Human immunodeficiency virus
ITT: Intention-to-treat
LTFU: Lost to follow up
MSM: Men who have sex with men
RCT: Randomized Controlled Trial
STD: Sexual transmitted disease
STI: Sexual transmitted infection
