# Supplementary material for: Online HIV prevention intervention on condomless sex among men who have sex with men: a web-based randomized controlled trial
Source: BMC Infect Dis. 2019 Jul 19;19:644. doi: 10.1186/s12879-019-4251-5 (PMC6642590; doi:10.1186/s12879-019-4251-5)
Supplement: Supplementary file 2 — Screenshots of the Scenario Experiencing Intervention. (DOCX 4707 kb) [file 12879_2019_4251_MOESM2_ESM.docx]

**Online HIV Prevention Intervention on Condomless Sex among Men Who Have Sex with Men: A Web-based Randomized Controlled Trial**

Weibin Cheng^1^*, Huifang Xu^1^*, Weiming Tang^2^, Fei Zhong^1^, Gang Meng^3^, Zhigang Han^1^, Ming Wang^1^, Jinkou Zhao^4^

Screenshots of the Scenario Experiencing Intervention.

| 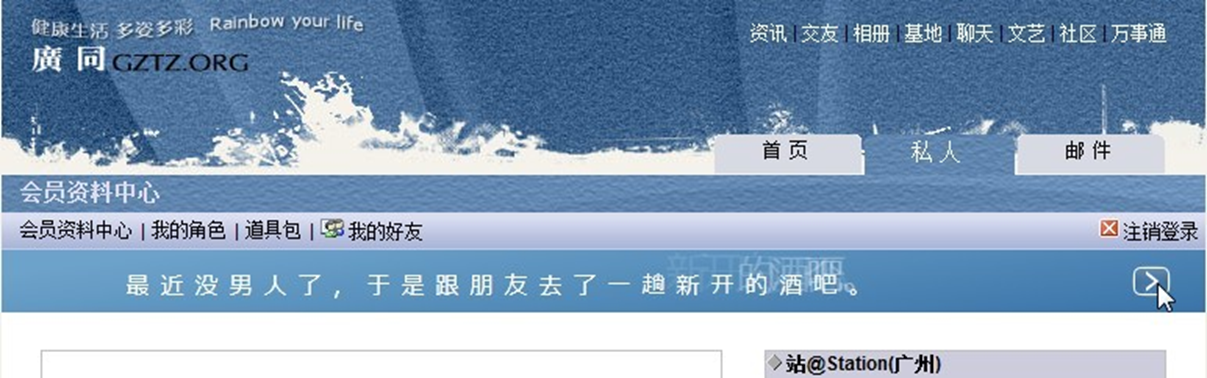  Being single for a while, I went to a bar with a friend….. |
| --- |
| 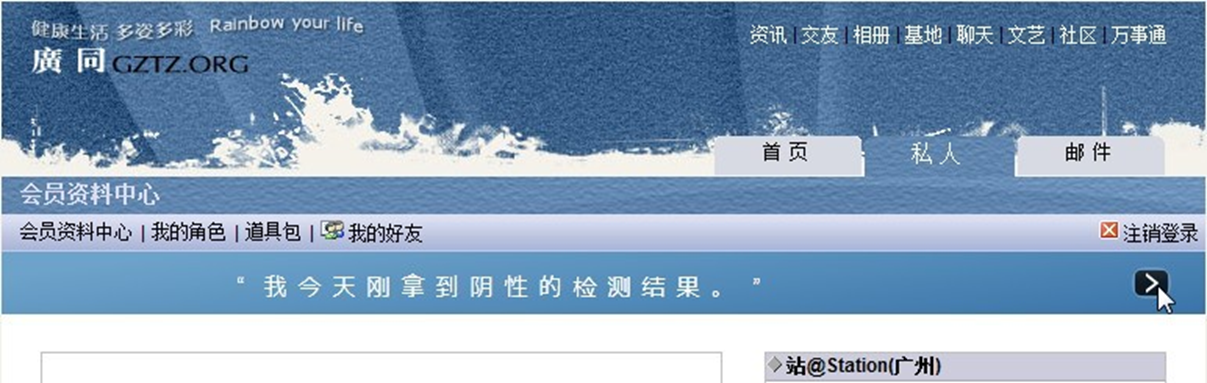  He said: “I get my test result today, it’s NEGATIVE.” |
| 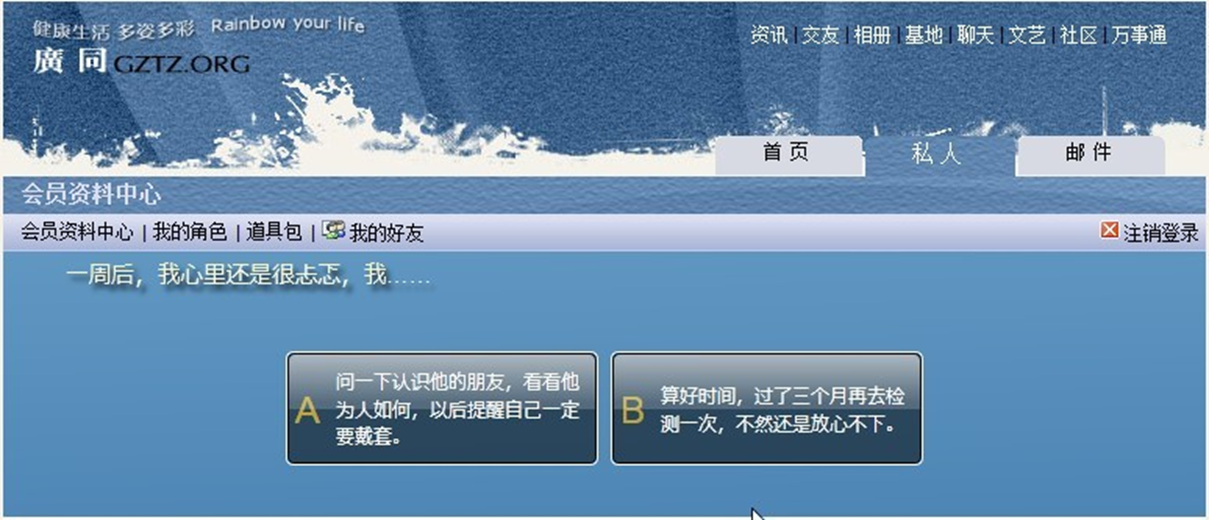  Count the days, and go to test after three months. Otherwise, I still cannot let go.  B  Ask his friend to find out what he is. And tell myself never do it again (have sex without condom).  A  A week passed, But I am still worried about it, I……  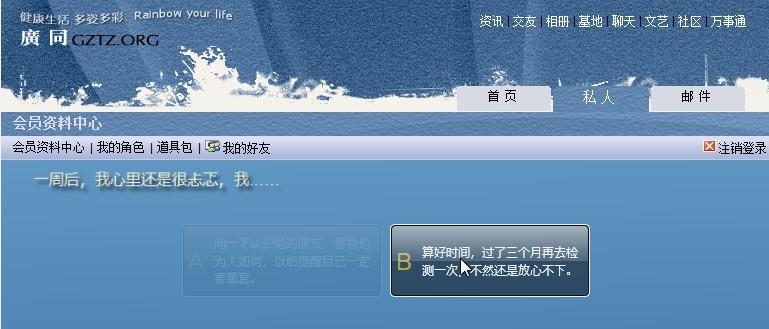  A week passed, But I am still worried about it, I……  Prompt: You choose B |
| 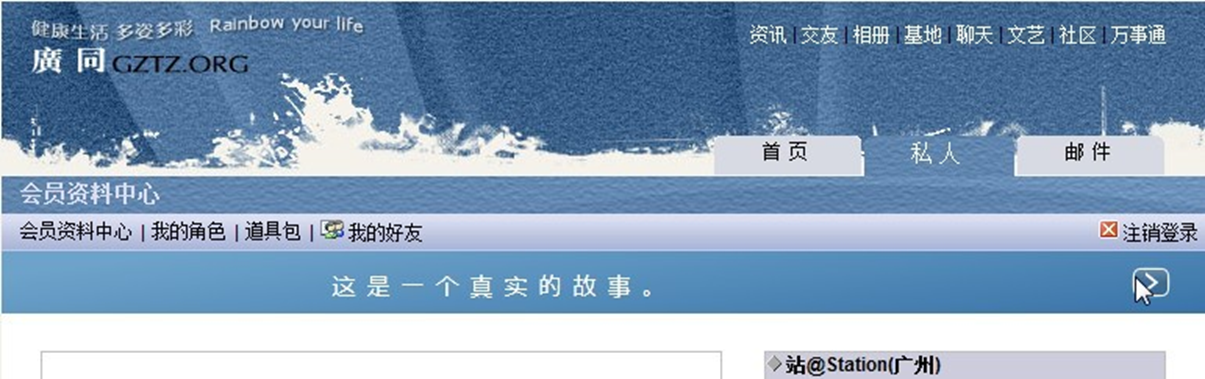  This is a true story of mine.  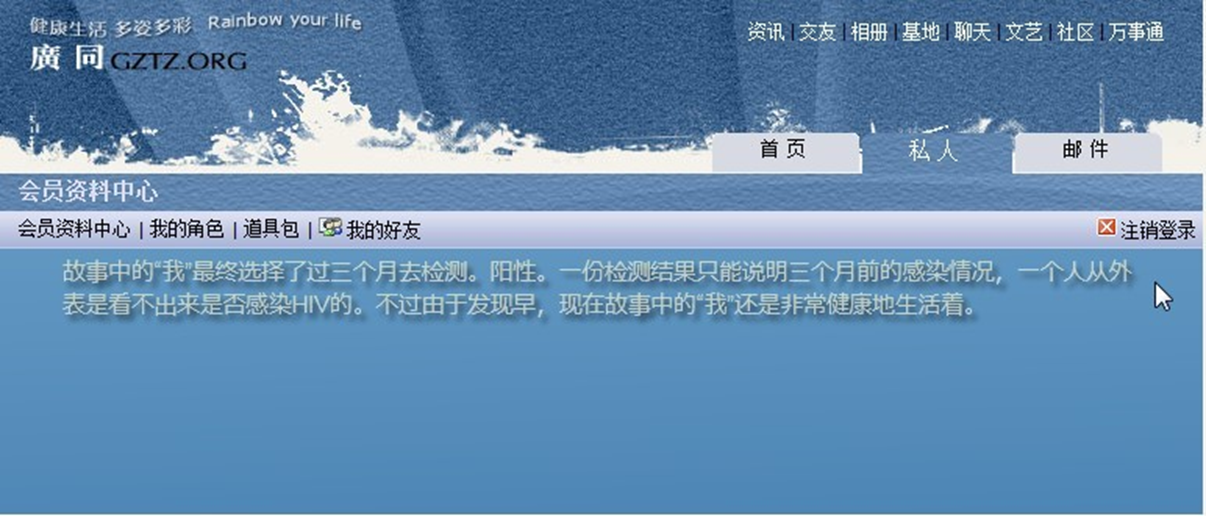  Finally, I went to test for HIV in three months, and result turned out to be POSITIVE. A negative test result cannot guarantee he is 100% safe for he might be infected in the “window period”. We cannot judge whether he is infected or not by their appearance. Fortunately, I knew my status in the very early stage, I can still live a healthy life.  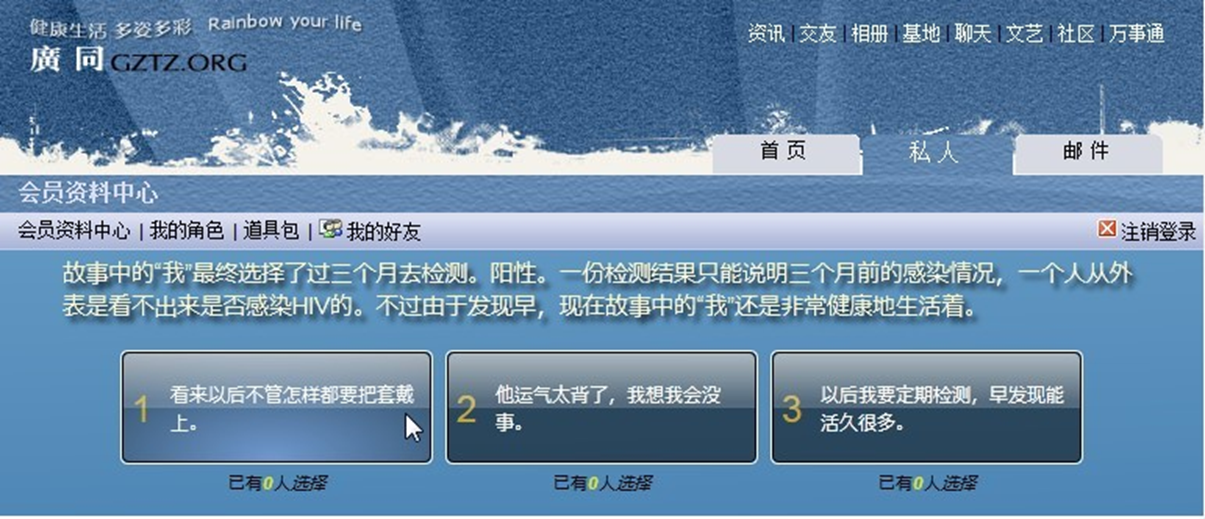  3  I will test HIV regularly.  He has no luck, I should be fine.  2  1  I will never do without a condom. |
