# Supplementary material for: Online HIV prevention intervention on condomless sex among men who have sex with men: a web-based randomized controlled trial
Source: BMC Infect Dis. 2019 Jul 19;19:644. doi: 10.1186/s12879-019-4251-5 (PMC6642590; doi:10.1186/s12879-019-4251-5)
Supplement: Supplementary file 3 — Screenshots of the Health Messenger. (DOCX 301 kb) [file 12879_2019_4251_MOESM3_ESM.docx]

**Online HIV Prevention Intervention on Condomless Sex among Men Who Have Sex with Men: A Web-based Randomized Controlled Trial**

Weibin Cheng^1^*, Huifang Xu^1^*, Weiming Tang^2^, Fei Zhong^1^, Gang Meng^3^, Zhigang Han^1^, Ming Wang^1^, Jinkou Zhao^4^

Screenshots of the **Health Messenger**

Theme I, named “know more & love yourself more”, delivered basic knowledge of HIV/AIDS and risky contact for HIV transmission


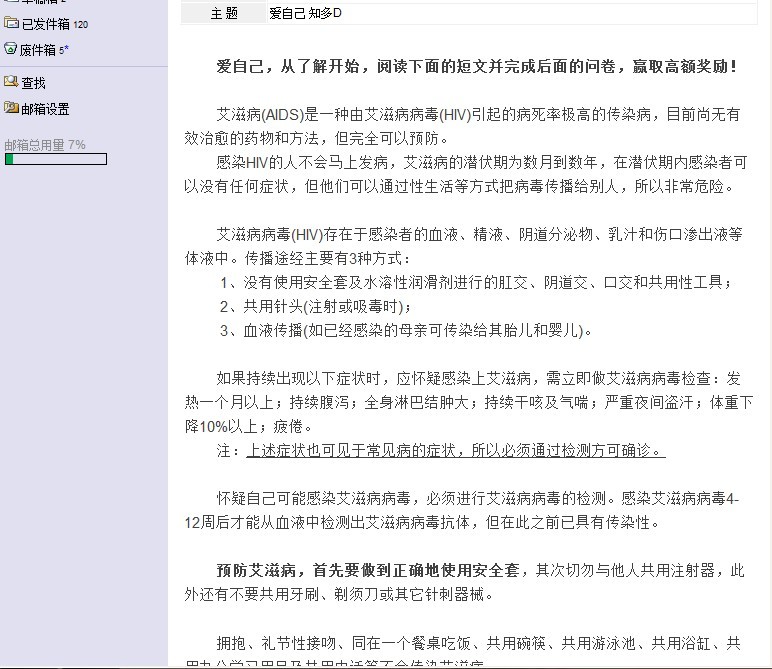


Script:

Love yourself, Start from understanding.

Read the following information and give out your answers to win the rewards.

AIDS is an infectious disease caused by HIV infection with a very high mortality rate. There is no cure for AIDS, but it can be easy to prevent.

HIV infected people does not develop to AIDS immediately. The incubation period of HIV infection last for several years. There can be no symptoms during the incubation period, but it can be transmitted to others through sexual contact. So it is very dangerous.

HIV contains in body fluids including blood, semen, vaginal secretions, breast milk and wound exudates. There are three main transmission routes:

1. Having sex intercourse (include anal sex and vaginal sex) without use condom and water-soluble lubricant.
2. Sharing needle
3. Vertical transmission (mother-to-child transmission)

If you persistent present the following symptoms, you should be aware of being infected and take HIV test ASAP: sustained fever for more than 1 month; persistent diarrhea; systemic lymphadenopathy; persistent dry cough and asthma; severe night sweats; weight loss of more than 10%; fatigue. Note: these symptoms also present in many common diseases, the only way to determine is to get an HIV test.

If you suspect that you are infected with HIV, you must take an HIV test. HIV antibodies can be detected from the blood after 4-12 weeks of infection.

To prevent AIDS, first make sure you use condoms correctly, then restraint of share syringes with others. Also, don't share toothbrush, razors, or other acupuncture instruments.

Theme II, named “risky domino, which one is you?”, released the latest local HIV epidemic data among MSM.
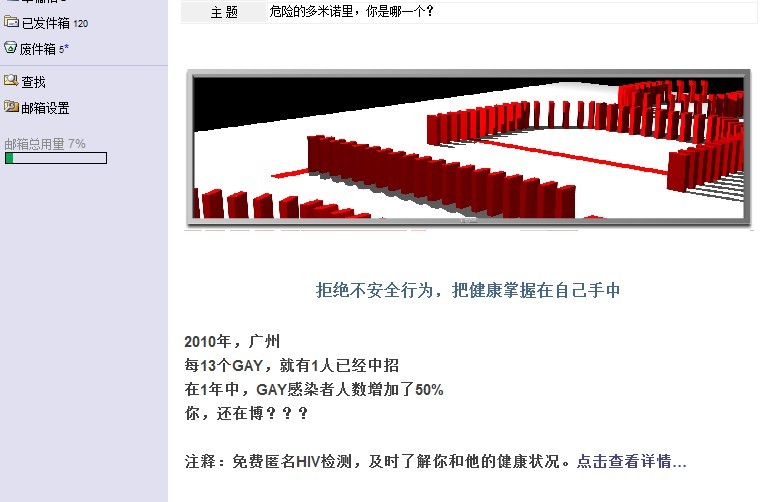


Script:

**Reject Unsafe Sex, Take Control your Health**

In 2010, Guangzhou

Every thirteen gay men there is an HIV positive.

In this year, gay men cases have increased by 50%.

Are you still risking???

Theme III, named “love faithfully & bottom safety”, clarified the misconceptions of sex behaviors, especially in intimate relationships.


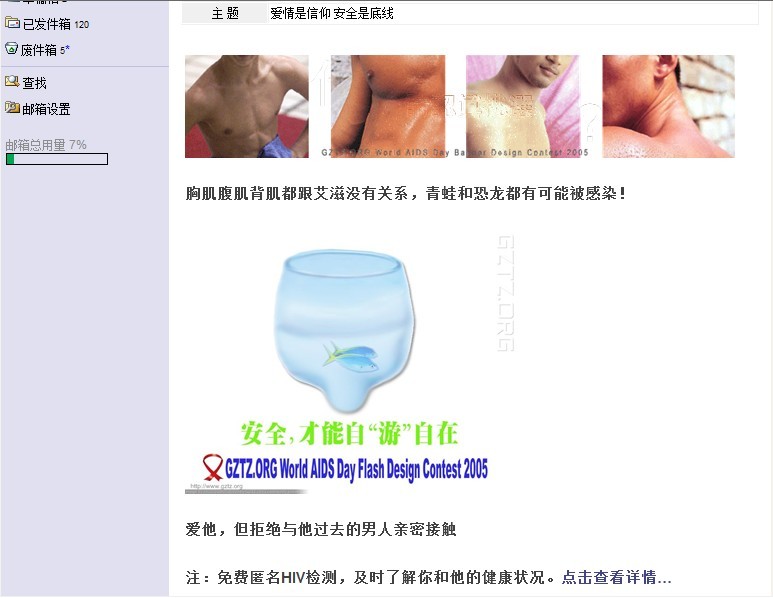


Script:

The chest muscles, abdominal muscles, and back muscles are all have nothing with AIDS. Both “frogs” and “dinosaurs” may be infected!

Love him but refuse to have any intimate contact with his previous partner/s.
