# Supplementary material for: Online HIV prevention intervention on condomless sex among men who have sex with men: a web-based randomized controlled trial
Source: BMC Infect Dis. 2019 Jul 19;19:644. doi: 10.1186/s12879-019-4251-5 (PMC6642590; doi:10.1186/s12879-019-4251-5)
Supplement: Supplementary file 4 — Comparison of participants’ baseline sociodemographic characteristics in completed records versus lost to follow-up and participants’ baseline and post-survey measurements on HIV/AIDS-related attitudes and behavioral intention in intervention group (n=501) versus control group. (DOCX 20 kb) [file 12879_2019_4251_MOESM4_ESM.docx]

**Online HIV Prevention Intervention on Condomless Sex among Men Who Have Sex with Men: A Web-based Randomized Controlled Trial**

Weibin Cheng^1^*, Huifang Xu^1^*, Weiming Tang^2^, Fei Zhong^1^, Gang Meng^3^, Zhigang Han^1^, Ming Wang^1^, Jinkou Zhao^4^

Table 1 Comparison of participants’ baseline sociodemographic characteristics in completed records versus lost to follow-up, 2011 (N=1100).

| **Characteristic** | **Completed record** (N=986) | **Lost to follow-up** (N=114) | ***P*-value**  (*χ*^2^ test) |
| --- | --- | --- | --- |
| Age –Year – no. (%) |  |  | 0.18 |
| ≤20 | 53 (5.4) | 3 (2.6) |  |
| 21-30 | 610 (61.9) | 77 (67.5) |  |
| 31-40 | 256 (26.0) | 31 (27.2) |  |
| ≥41 | 67 (6.8) | 3 (2.6) |  |
| Educational – no. (%) |  |  | 0.69 |
| Junior high school or below | 33 (3.3) | 4 (3.5) |  |
| Senior High school | 170 (17.2) | 16 (14.0) |  |
| College or above | 783 (79.4) | 94 (82.5) |  |
| Annual income (US dollar) |  |  | 0.12^§^ |
| No income | 83 (8.4) | 3 (2.6) |  |
| Less than $5,351 | 328 (33.3) | 36 (31.6) |  |
| $5,351 to 12,485 | 387 (39.2) | 53 (46.5) |  |
| $12,485 or above | 188 (19.1) | 22 (19.3) |  |
| Ethnicity – no. (%) |  |  | 0.76^§^ |
| Han | 958 (97.2) | 112 (98.2) |  |
| Minority | 28 (2.8) | 2 (1.8) |  |
| Marital status – no. (%) |  |  | 0.06 |
| Currently married | 114 (11.6) | 20 (17.5) |  |
| Single^+^ | 872 (88.4) | 94 (82.5) |  |
| Sexual orientation – no. (%) |  |  | 0.17 |
| Homosexual | 774 (78.5) | 83 (72.8) |  |
| Bisexual/heterosexual/others | 212 (21.5) | 31 (27.2) |  |
| Places of meeting sex partner – no. (%) |  |  | 0.31 |
| Internet | 871 (88.3) | 97 (85.1) |  |
| Others | 115 (11.7) | 17 (14.9) |  |
| Perceived of HIV epidemic among MSM– no. (%) |  |  | 0.84 |
| Nothing serious | 345 (35.0) | 41 (36.0) |  |
| Serious/very serious | 641 (65.0) | 73 (64.0) |  |
| HIV Tested in the previous six months – no. (%) |  |  | 0.29 |
| Yes | 241 (24.4) | 33 (29.9) |  |
| No | 745 (75.6) | 81 (71.1) |  |
| Had Anal sex with male in the previous three months |  |  | 0.93 |
| Yes | 740 (75.1) | 86 (75.4) |  |
| No | 246 (24.9) | 28 (24.6) |  |
| Group sex with male in the previous three months |  |  | 0.35 |
| Yes | 125 (12.7) | 18 (15.8) |  |
| No | 861 (87.3) | 96 (84.2) |  |
| Condomless sex with male in the previous three months |  |  | 0.51 |
| Yes | 452 (45.8) | 56 (49.1) |  |
| No | 534 (54.2) | 58 (50.9) |  |
| Condomless sex with male regular partner/s in the previous three months |  |  | 0.09 |
| Yes | 354 (53.1) | 51 (63.0) |  |
| No | 313 (46.9) | 30 (37.0) |  |
| Condomless sex with male casual partner/s in the previous three months |  |  | 0.08 |
| Yes | 185 (34.3) | 29 (45.3) |  |
| No | 355 (65.7) | 35 (54.7) |  |

§ Fisher’s Exact Test

+ Included divorced and widowed.

Table 2 Comparing participants’ baseline and post-survey measurements on HIV/AIDS-related attitudes and behavioral intention in intervention group (n=501) versus control group (n=485).

|  | **Control group** (%) | **Online Intervention** (%) | ***P*-value**  (*χ*^2^ test) |
| --- | --- | --- | --- |
| Perceived of HIV epidemic among MSM (serious/very serious) |  |  |  |
| Baseline | 64.7 | 65.3 | 0.86 |
| Post-survey | 69.3 | 75.0 | **0.04** |
| Knowing where to take HIV antibody test |  |  |  |
| Baseline | 86.4 | 86.0 | 0.87 |
| Post-survey | 88.0 | 93.0 | **0.01** |
| Perceived of taking HIV antibody test in the next 3 months(certainly) |  |  |  |
| Baseline | 24.1 | 23.4 | 0.78 |
| Post-survey | 29.1 | 44.3 | **<0.00** |
| Having multiple male sex partners in last 3 months |  |  |  |
| Baseline | 41.4 | 39.3 | 0.50 |
| Post-survey | 41.6 | 35.5 | **0.04** |
| Having group sex in the last 3 months |  |  |  |
| Baseline | 14.0 | 11.4 | 0.21 |
| Post-survey | 10.7 | 6.6 | **0.02** |
| Having condomless anal intercourse in the last anal sex |  |  |  |
| Baseline | 31.7（375） | 26.3（365） | 0.10 |
| Post-survey | 26.0（369） | 12.0（350） | **<0.00** |
